# Effect of baicalin on eradicating biofilms of bovine milk derived *Acinetobacter lwoffii*

**DOI:** 10.1186/s12917-024-04015-w

**Published:** 2024-05-20

**Authors:** Chengjun Ma, Cui Mei, JingJing Liu, Hui Li, Min Jiao, Huiming Hu, Yang Zhang, Jing Xiong, Yuzhang He, Wei Wei, Hongzao Yang, Hongwei Chen

**Affiliations:** 1 College of Veterinary Medicine, Southwest University, Chongqing, 402460 China; 2 National Center of Technology Innovation for Pigs, Chongqing, 402460 China; 3 Present Address: Immunology Research Center, Medical Research Institute, Southwest University, Chongqing, 402460 China; 4 Chongqing Academy of Animal Sciences, Chongqing, 402460 China

**Keywords:** Baicalin, *Acinetobacter lwoffii*, Biofilm, Eradication, Trehalose

## Abstract

**Background:**

*Acinetobacter lwoffii* (*A.lwoffii*) is a serious zoonotic pathogen that has been identified as a cause of infections such as meningitis, bacteremia and pneumonia. In recent years, the infection rate and detection rate of *A.lwoffii* is increasing, especially in the breeding industry. Due to the presence of biofilms, it is difficult to eradicate and has become a potential super drug-resistant bacteria. Therefore, eradication of preformed biofilm is an alternative therapeutic action to control *A.lwoffii* infection. The present study aimed to clarify that baicalin could eradicate *A.lwoffii* biofilm in dairy cows, and to explore the mechanism of baicalin eradicating *A.lwoffii.*

**Results:**

The results showed that compared to the control group, the 4 MIC of baicalin significantly eradicated the preformed biofilm, and the effect was stable at this concentration, the number of viable bacteria in the biofilm was decreased by 0.67 Log_10_CFU/mL. The total fluorescence intensity of biofilm bacteria decreased significantly, with a reduction rate of 67.0%. There were 833 differentially expressed genes (367 up-regulated and 466 down-regulated), whose functions mainly focused on oxidative phosphorylation, biofilm regulation system and trehalose synthesis. Molecular docking analysis predicted 11 groups of target proteins that were well combined with baicalin, and the content of trehalose decreased significantly after the biofilm of *A.lwoffii* was treated with baicalin.

**Conclusions:**

The present study evaluated the antibiofilm potential of baicalin against *A.lwoffii*. Baicalin revealed strong antibiofilm potential against *A.lwoffii*. Baicalin induced biofilm eradication may be related to oxidative phosphorylation and TCSs. Moreover, the decrease of trehalose content may be related to biofilm eradication.

**Supplementary Information:**

The online version contains supplementary material available at 10.1186/s12917-024-04015-w.

## Background

Following with the continuous development of dairy farming in China, the incidence of mastitis in dairy cows is also on the rise. Infections associated with biofilms of *Klebsiella pneumoniae* and *Acinetobacter baumannii* are the main culprits for the persistent mastitis in dairy cows [1, 2]. In recent years, the infection rate and detection rate of *A.lwoffii* have been increasing. Its resistance to penicillins and cephalosporins can reach more than 50%, showing a phenomenon of multiple drug resistance, and the resistance rate is rising rapidly [3]. Biofilm is widely recognized as the dominant mode of bacterial growth in nature [4]. Bacterial biofilms are surrounded by extracellular polymeric substances (EPS), which act as diffusion barriers to inhibit the deep penetration of traditional antibiotics [5]. The major components of EPS are exopolysaccharides (which are sometimes called EPSs), proteins, extracellular DNA, lipids and other biopolymers [6]. It has been extensively reported that the tolerance of biofilms to various antibiotics is 10–1000 times greater than that of planktonic cells [4, 5, 7]. The formation of bacterial biofilm is one of the important resistance mechanisms. Due to the existence of biofilms, which are not easily eradicate, and has now become a potential super drug-resistant bacteria [8]. In addition, problems such as antibiotic residues in cows and milk seriously affect human health and the vigorous development of animal husbandry. Therefore, it is of great significance to develop efficient and low-toxicity drugs to eradicate *A.lwoffii* biofilm.

Traditional Chinese medicine (TCM), which originated in ancient China, has been widely used for prevention and treatment of various diseases in China and other countries [9]. TCM and its individual components have unique advantages in the prevention and treatment of infectious diseases, including treating the symptoms and root causes, low likelihood of drug resistance, low cost, and rich action sites. Baicalin (5,6,7-trihydroxyflavone) is one of the major flavonoid monomers purified from the roots of Scutellaria baicalensis, which is listed as a Chinese herbal medicine in the Chinese Pharmacopoeia. Numerous TCM formulae containing baicalin are widely used clinically for treating fever, bronchitis and upper respiratory tract infections [10–13]. Baicalin alone or in combination with other antibacterial agents can inhibit the biofilm formation [14, 15]. However, we rarely found the effect of baicalin on the biofilm of *A.lwoffii* in the literature, especially the biofilm eradication experiment. Considering the high economic value of dairy cows and the inadvisability of using antibiotics during lactation, our team combined literature and previous research to find that baicalin can significantly eradicate the preformed biofilm of *A.lwoffii* within 24 h, with an eradication rate of up to 50%. This work intends to combine the phenotype of *A.lwoffii* preformed biofilm with RNA-seq technology to analyze its gene expression level and initially clarify its mechanism of action at the molecular level, which helped to lay a theoretical foundation for developing baicalin as a potential biofilm eradication agent.

## Results

### Baicalin significantly reduced the biomass and viable bacteria of biofilms in *A.lwoffii*

**Fig. 1:**
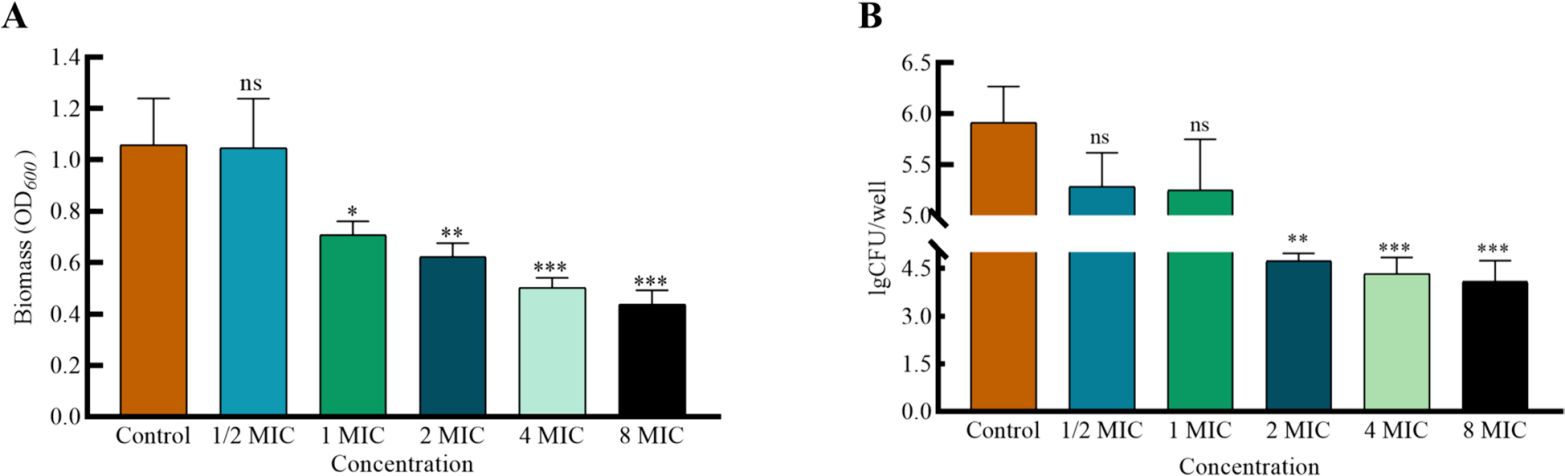
Effect of baicalin on *A.Lwoffii* biofilm. *A.Lwoffii* biofilms were cultured for 24 h at 37 °C in 96-well plates. Post-washing, these biofilms were exposed to baicalin for 3 h. Biomass quantification was conducted using crystal violet (CV) staining (**A**) and viable bacteria of biofilm by colony counting (**B**). The significance of results was analyzed using an unpaired two-tailed t-test. Notably, * *P* < 0.05 and ** *P* < 0.01 when compared to the control group

MIC (minimum inhibitory concentration) on *A.lwoffii* for baicalin expressed in 2048 μg/mL. The crystal violet assay represents the biomass of *A.lwoffii*. The results showed that baicalin exhibited a more significant effect on reducing the biomass of *A.lwoffii* biofilms, with a reduction rate of 41.28% at 8 MIC. We found that baicalin reduced biomass in a dose-dependent manner, while 1/2 MIC baicalin had almost no effect in *A.lwoffii* (Fig. 1A). In addition, the viability of bacteria in the biofilm was affected by baicalin. At 8 MIC, baicalin had a significant killing rate (> 90%) of bacteria in the biofilm (Fig. 1B). Considering that the higher the drug concentration, the greater the adverse reactions in vivo and in vitro studies, this experiment finally choose concentration of 4 MIC (8192 μg/mL) for subsequent experimental research.

### Baicalin eradicated biofilm of *A.lwoffii*

**Fig. 2:**
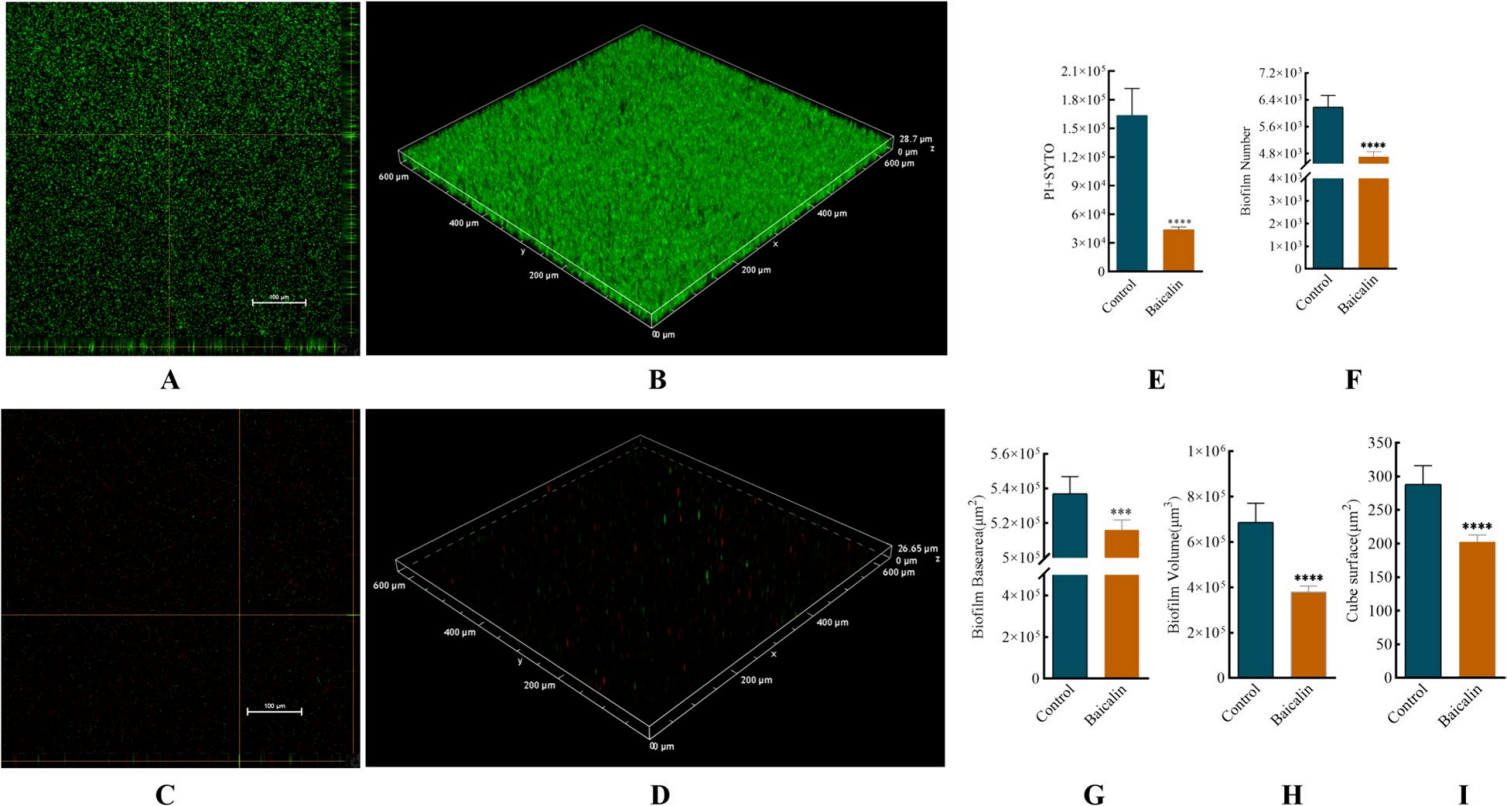
CLSM analysis of baicalin treated *A.lwoffii* pre-biofilms. *A.Lwoffii* biofilm was formed for 24 h at 37 °C on chambered coverglass slides. After washing, biofilms were treated with baicalin for 3 h at 37 °C as described and subsequently stained with SYTO 9 and PI for 20 min in the dark. Images were acquired by CLSM. Using BiofilmQ software for image processing.. **A** and **B** 3D and orthogonal views biofilm representation in the objective of 20X in the control group. **C** and **D** 3D and orthogonal views biofilm representation of baicalin in the objective of 20X. **E **total fluorescence intensity of biofilms (PI + SYTO9). **F** represents the number of biofilms. **G** bottom area of biofilms. **H** represents the volume of biofilms. **I** represents the surface area of biofilms. Unpaired t-test (two-tailed) was used to measure statistical significance. * *P* < 0.05, ** *P* < 0.01 compared with the control group

To corroborate the anti-biofilm activity of baicalin, confocal laser scanning microscopy (CLSM) was used to visualize the preformed biofilm after treatment with baicalin. Biofilms were grown for 24 h and then treated with baicalin for an additional 3 h, followed by staining with SYTO 9 (live) and propidium iodide (PI) (dead) bacteria. Representative orthogonal views of Z-stacks and 3D images (Fig. 2A–D) showed dead bacteria (red) and less dense structures in baicalin-treated biofilms. The results showed that baicalin markedly decreased the total fluorescence intensity of living (SYTO 9) and dead (PI) bacteria by 67% (Fig. 2E) at the concentration of 8192 μg/mL. Furthermore, the number (24.62%), area (3.89%) and volume (44.74%) of *A.Lwoffii* biofilms were decreased (Fig. 2F–H). In addition, after baicalin treatment, the ratio of fluorescence intensity per unit area to biofilm surface area decreased (F ig. 2I).

### Analyzing the correlation of differential gene expression with RNA-seq technology

**Fig. 3:**
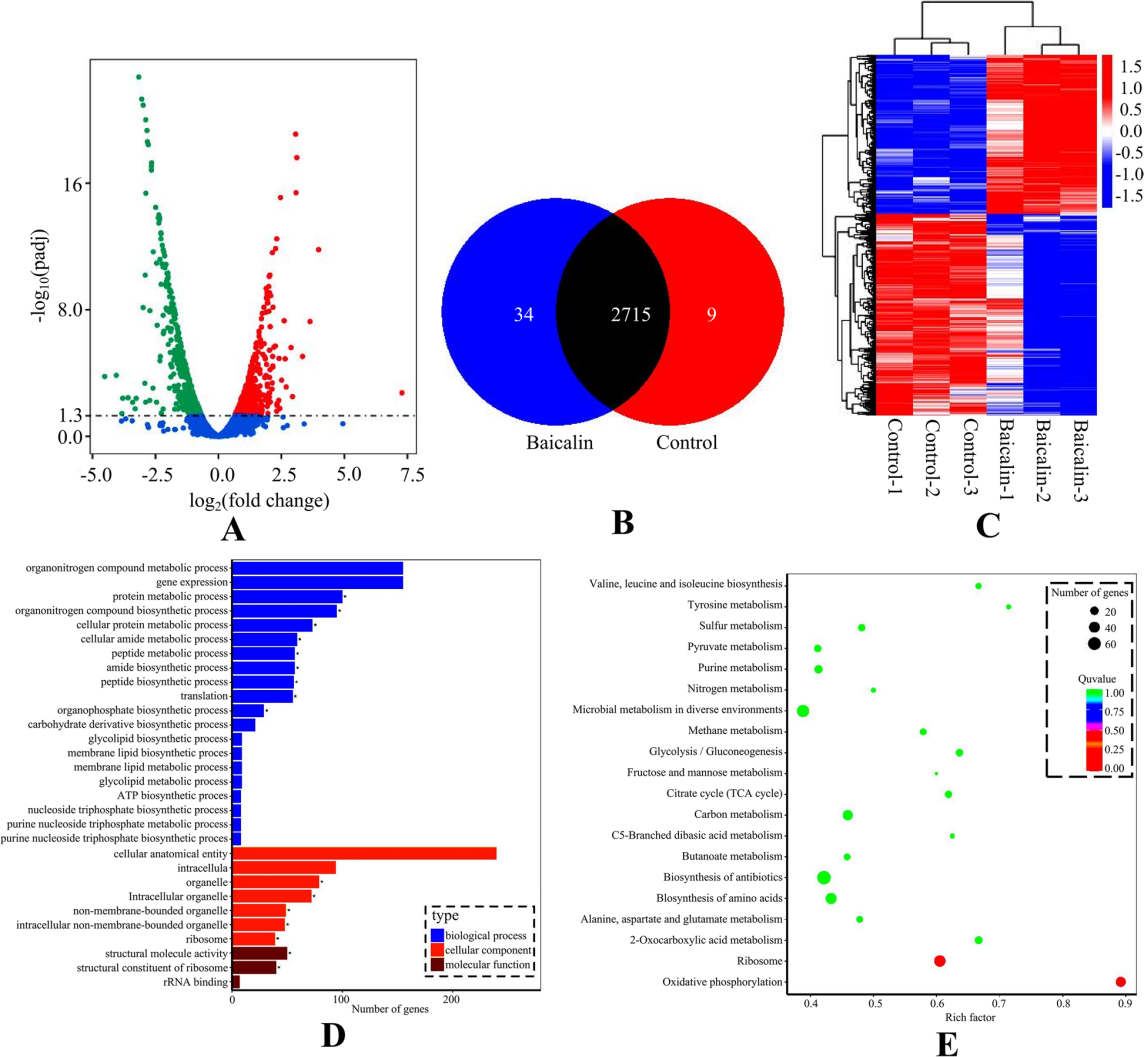
Analysis of differential gene expression. The preformed biofilm was treated with 4 MIC baicalin for 3 h, and the upper culture medium in cell culture flasks was collected and centrifuged. The supernatant was taken and frozen in liquid nitrogen for storage. There were 6 samples in both the baicalin group and the control group. The samples were analyzed by LC–MS, and the data were extracted and preprocessed using Mas-terView (SCIEX). **A** Volcano plots showed the fold change of differential gene in baicalin group vs control group. The green dots represent significantly down-regulated genes (466), the red dots represent significantly up-regulated genes (367), and grey dots represent non-significantly changed differential genes. **B** Venn diagram. **C** Cluster analysis diagram of differentially expressed genes, the twenty-four hours *A.Lwoffii* pre-biofilms was treated with baicalin (4 MIC) for 3h, and the untreated biofilms was used as the control. All experiments were performed at least in triplicate, and there were 6 samples in both the baicalin group and the control group. **D** GO annotation enrichmentan analysis diagram and (**E**) KEGG enrichment analysis diagram. The* p* value is the significance of enrichment of this metabolic pathway. The ordinate is the name of the metabolic pathway, the abscissa is the rich factor. The larger the rich factor, the more metabolites enriched in the pathway. The size of the dots represents the number of differential metabolites enriched into the pathway

According to the differential gene screening criteria (qvalue < 0.05), and these differences are visualized in the volcano plot (Fig. 3A). In this plot, green dots denote genes were down-regulated (466 in total), red dots indicated up-regulated genes (367 in total), while blue dots symbolized genes with no significant changes. Subsequently, the identified genes were plotted into a Venn diagram, and the results showed that there were 2715 differentially expressed genes, including 9 unique differential genes in the control group and 34 unique differential genes in the drug group (Fig. 3B). Hierarchical clustering analysis was performed using FPKM values as expression levels (using Log10(FPKM + 1) values), and different colors represented different experimental groups. The results showed that in the control group genes clustered into one group, and genes in the drug group clustered into one group (Fig. 3C). In this work, GO software was used for analysis, and the top 30 most significantly enriched functional groups were obtained. The results showed that 20 were involved in biological processes, 7 in cellular processes, and 3 in molecular functions, mainly focusing on oxidative phosphorylation, biofilm regulation systems, and alginate synthesis (Fig. 3D). Pathway analysis of differential metabolites was performed using the Kyoto Encyclopedia of Genes and Genomes (KEGG) database, the top 20 metabolic pathways based on *p*-value were chosen for examination the alterations of total KEGG metabolic pathway (Fig. 3E). In order to sort out the relationship between differentially expressed genes and biofilms, combined with the correlation of phenotype analysis results in previous research. Classification and sorting were carried out from two-component regulatory systems, virulence factors, sugar metabolism and other regulatory pathways. The results showed that there were 26 significantly different representative genes, and the function relevance of the genes were described as follows (Table 1).

**Table 1:** Baicalin intervention genes related to the biofilm of *A.lwoffii*

NO	Gene ID	Gene	Product	log2 |Fold Change|	P-agjust
1	02640	*ahpC*	Alkyl hydroperoxide reductase C	7.2832	1.78 × 10^–3^
2	01420	*betL*_3	HTH-type transcriptional regulator BetI	3.9768	1.61 × 10^–12^
3	01069	*sodB*_1	Superoxide dismutase [Mn/Fe]	3.3389	8.92 × 10^–12^
4	00418	*hmp*_1	Flavohemo protein	3.0815	4.05 × 10^–16^
5	00417	*nsrR*	HTH-type transcriptional repressor NsrR	3.0652	7.95 × 10^–20^
6	01421	*betB*	NAD/NADP-dependent betaine aldehyde dehydrogenase	2.881	2.43 × 10^–6^
7	01773	*clpB*	Chaperone protein ClpB	2.4656	8.14 × 10^–16^
8	02952	*mprA*	Response regulator MprA	2.4094	1.19 × 10^–5^
9	02669	*sdpR*	Transcriptional repressor SdpR	2.3932	5.30 × 10^–3^
10	00867	*rhtC*_2	Threonine efflux protein	2.3209	8.94 × 10^–3^
11	02960	*blC*_3	Outer membrane lipoprotein Blc	2.2581	1.94 × 10^–6^
12	00618	*ppiC*	Peptidyl-prolyl cis–trans isomerase C	2.1985	2.33 × 10^–6^
13	02956	*ufaA*1	Tuberculostearic acid methyltransferase UfaA1	2.1685	6.93 × 10^–6^
14	02086	*otsA*	Trehalose-6-phosphate synthase	2.1662	4.15 × 10^–5^
15	00619	*mutM*	Formamidopyrimidine-DNA glycosylase	2.1556	3.53 × 10^–5^
16	01763	*liuE*	3-hydroxy-3-isohexenylglutaryl-CoA/hydroxy-methylglutaryl-CoA lyase	2.0776	1.29 × 10^–9^
17	02555	*htpG*	Chaperone protein HtpG	2.0441	6.41 × 10^–11^
18	01452	*fumC*	Fumarate hydratase class II	2.0231	1.87 × 10^–4^
19	02085	*otsB*	Trehalose-6-phosphate phosphatase	2.0008	1.96 × 10^–3^
20	00105	*tnsA*	Transposon Tn7 transposition protein TnsA	1.9409	3.70 × 10^–9^
21	00764	*lolA*	Outer-membrane lipoprotein carrier protein	1.9001	1.81 × 10^–9^
22	02286	*rcsC_3*	Sensor histidine kinase RcsC	1.7063	3.62 × 10^–9^
23	00450	*mymA*	Putative FAD-containing monooxygenase MymA	1.5207	1.27 × 10^–6^
24	02799	*yidC*	Membrane protein insertase YidC	-2.0796	1.37 × 10^–11^
25	02566	*rplJ*	50S ribosomal protein L10	-2.3429	2.05 × 10^–14^
26	02565	*rplL*	50S ribosomal protein L7/L12	-2.3674	1.02 × 10^–14^

### qRT-PCR is used to detect the transcription level of differential genes

**Fig. 4:**
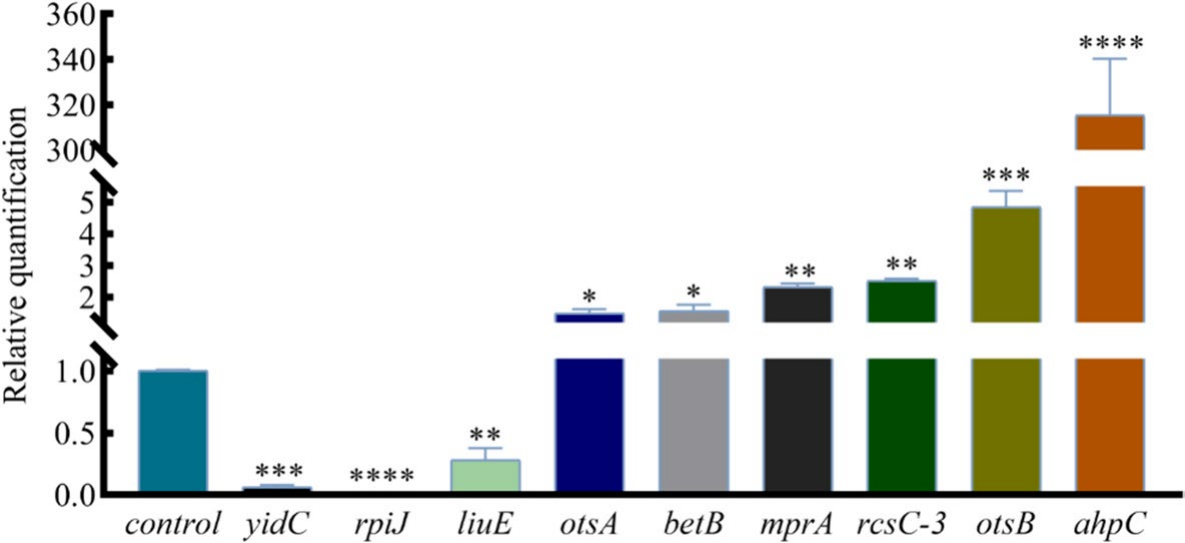
Fluorescence quantitative PCR results of differential genes. Effects of 4 MIC baicalin on the regulated genes *ahpC*, *otsB*, *rcsC-3*, *mpaA*, *betB*, *otsA*, *liuE*, *rpiJ* and *yidC* in *A.Lwoffii* biofilms

To verify the accuracy of RNA-Seq results, we selected the genes in Table 3 that were most relevant to this study, and used qRT-PCR to detect the transcription level of differential genes. The results showed that there were 3 significantly down-regulated genes (*yidC*,*rpiJ*, *liuE*) and 6 significantly up-regulated genes (*otsA*, *betB*, *mprA*, *rcsC-3*, *otsB*, *ahpC*) (Fig. 4). Among them, *ahpC* had the highest fold increase, which was consistent with the highest fold increase in transcriptome expression level. In summary, the trends of 8 genes were consistent with the transcriptome results, and the inconsistent gene was *liuE*. This indicates that the data generated by RNA-seq can be used to further study the expression of specific genes.

### Molecular docking of these proteins with active ingredient

**Fig. 5:**
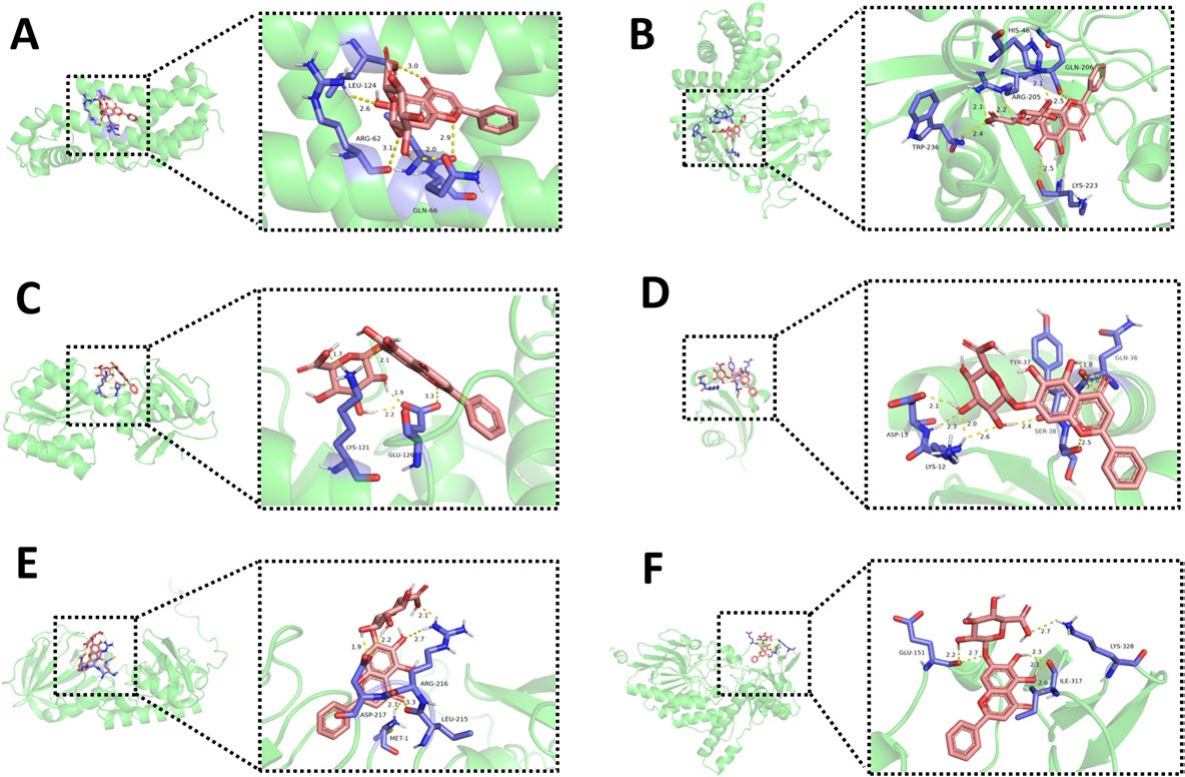
Three-dimensional interaction between baicalin and target proteins. AutoDockTools 1.5.7 software was applied to process proteins. PyMOL analyze the binding situation of receptor proteins and ligands in molecular docking, and it can mark hydrogen bonds. The top 6 binding force compounds were imported into PyMOL for further visualization. **A** Baicalin-betI_3, (**B**) Baicalin-hmp-1, (**C**) Baicalin-mprA, (**D**) Baicalin-ppiC, (**E**) Baicalin-mutM, (**F**). Baicalin-mymA. The yellow dotted line indicates the hydrogen bond distance or π-stacking distance, while the active ingredient is shown in red

Autodock software was used to dock the active ingredient (baicalin) with 26 proteins related to the *A.lwoffii* biofilm at the molecular level. After 26 groups of target proteins and compound molecule were introduced into AutoDock Vina, the affinity value of the optimal binding postures were calculated (the results is shown in Table 2). The more stable the binding between the ligand and the receptor, the average value of 26 groups of affinity was − 4.01 kcal/mol, and there are 11 groups with affinity ≤ -4.25 kcal/mol. The docking score indicates that baicalin may affect *A.Lwoffii* biofilm by regulating proteins. Figure 5 showed the docking results of baicalin with top 6 of affinity absolute value for target proteins (betL-3, hmp-1, nsrR, mprA, ppiC, mutM, mymA).

**Table 2:** Molecular docking results of baicalin active ingredient and targets

Active ingredient	Targets	Affinity/(kcal/mol)	Active ingredient	Targets	Affinity/(kcal/mol)
Baicalin	ahpC	-1.69	Baicalin	otsA	-2.95
	betL-3	-5.34		mutM	-5.67
	sodB-1	-1.64		liuE	-4.11
	hmp-1	-7.28		htpG	-2.05
	nsrR	-4.87		fumC	-3.18
	betB	-3.47		otsB	-2.97
	clpB	-2.48		tnsA	-4.36
	mprA	-6.68		lolA	-2.78
	sdpR	-4.66		rcsC-3	-3.49
	rhtC-2	-4.65		mymA	-5.02
	blC-3	-3.95		yidC	-3.25
	ppiC	-5.92		rplJ	-4.73
	ufaA1	-4.05		rplL	-2.92

### The content of trehalose significantly decreased after the biofilm of *A.lwoffii* was treated with baicalin

**Fig. 6:**
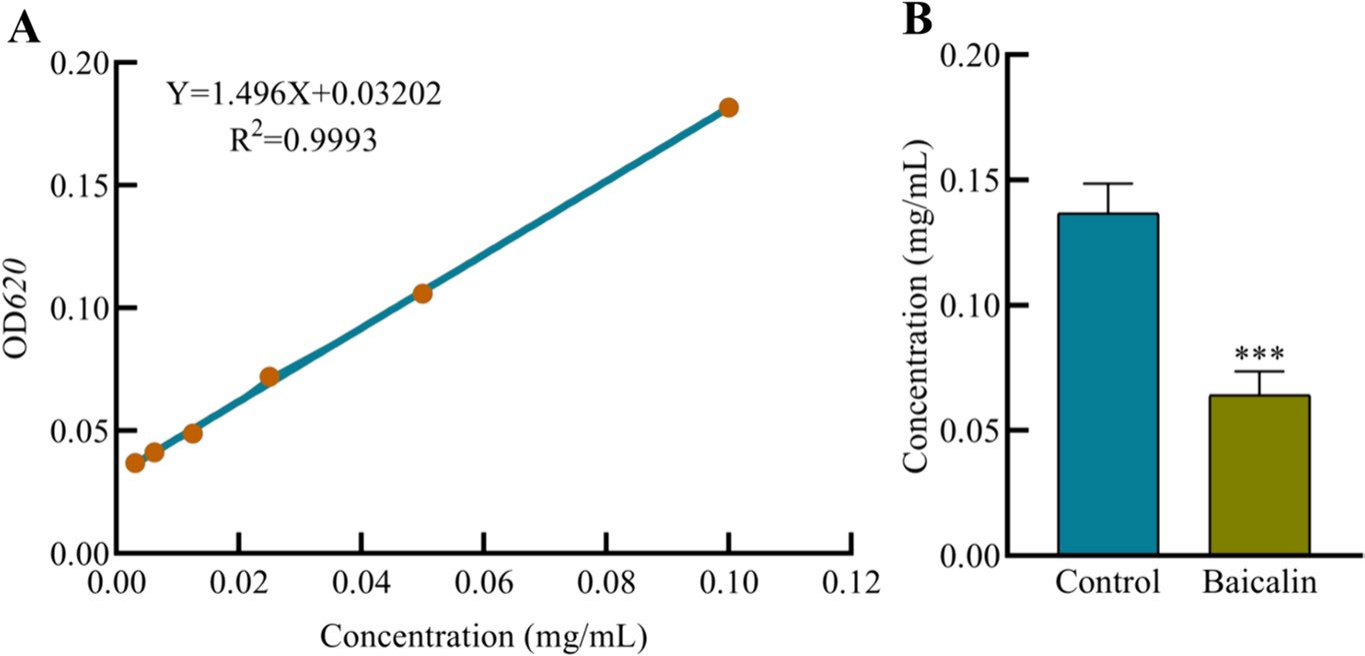
Standard curve and content determination of trehalose. Sodium alginate standard product was diluted with ddH_2_O water at a ratio, with concentrations of 0.1, 0.05, 0.025, 0.0125, 0.00625, 0.003125 mg/mL respectively. Take 0.25 mL of standard solution and 1 mL of working solution to an EP tube, water bath at 95 °C for 10 min, naturally cool to room temperature, take the solution to the colorimetric dish, measure *OD*_*620*_nm. Calculate the concentration of ΔA standard (**A**) and using the formula obtained from the drawn standard curve to calculate X value can measure the trehalose content of each group (**B**). Unpaired t-test (two-tailed) was used to measure statistical significance. * *P* < 0.05, ** *P* < 0.01 compared with the control group

With trehalose content as the y-axis and the standard product concentration as the x-axis, a standard curve is drawn (Fig. 6A). Linear regression analysis gives the equation: Y = 1.1496X + 0.0692, *R*^*2*^ = 0.9993. When the trehalose concentration is between 0.003125 and 0.1 mg/mL, the absorbance gradually increased with the increase of trehalose concentration. The concentration of trehalose was positively correlated with the absorbance, indicating that there was a good linear relationship between trehalose and absorbance. According to the standard curve and measurement method, the absorbance value of the sample solution collected after baicalin acted on the biofilm of *A.lwoffii* was measured. The concentration of trehalose in the test product (mg/mL) based on the trehalose standard curve was calculated. Compared with the control group, it showed that the content of trehalose in the drug group significantly decreased (Fig. 6B), indicating that the content of trehalose decreased after baicalin acted on the biofilm of *A.lwoffii*, leading to the eradication of biofilm cells.

## Discussion

The genus Acinetobacter is ubiquitous in nature and is frequently found in soil, water and dry environments [16]. In recent years, the infection of *A.lwoffii* in medical clinics and livestock has been quite serious, and it is easy to cause bovine acute interstitial pneumonia, but it has not attention has been paid to it [17]. The development speed of antibiotics has fallen far behind the evolution speed of bacterial resistance, and various resistance mechanisms have also increased the difficulty of antibiotic development [18]. One of the most significant characteristics of biofilms is lower susceptibility to antibiotic treatments and the host immune response [19, 20]. The infection rate of multi-drug resistant Gram-negative bacteria has been on the rise, and the biofilm formation is one of its important resistance mechanisms, so it is urgent to actively develop drugs for treating biofilms [21]. Baicalin has a promising antibacterial effect [22–24], MIC is 2048 μg/mL, and it has been proved that it can inhibit the formation of biofilm [10, 14, 25]. Combined with previous research, it was found that baicalin could significantly eradicate the preformed biofilm of *A.lwoffii* in the milk of cows. Therefore, combining phenotype and data analysis to study the mechanism of biofilm formation will be conducive to better clinical effects.

To the best of our knowledge, this work presented the first evidence that baicalin eradication in the *A.lwoffii* biofilm from dairy. Based on the results of biomass and biofilm bacterial assays, we concluded that baicalin can significantly reduce biomass and viable bacteria of biofilms in *A.lwoffii*. The above findings were further supported by CLSM findings. In summary, this indicates that baicalin has a significant eradicating effect on preformed biofilms of *A.lwoffii*. In addition, some studies have shown that baicalin with a certain concentration can eradicate mature biofilm of bacteria [26, 27].

RNA-seq has become the preferred technology for gene analysis, which can improve the understanding of gene expression and the complexity of corresponding protein regulation. Many proteins have been proven to participate in biofilm formation, promoting or inhibiting biofilm formation by interacting with bacterial biofilm-forming proteins [27, 28]. For example, *ahpC* (alkyl hydroperoxide reductase) significantly affects the biofilm formation of *Campylobacter jejuni*, where Campylobacter oxidative stress regulator (cosR) is an important reaction regulator. ahpC is positively regulated, thereby alleviating oxidative stress of aggregates by reducing the ability of *Campylobacter jejuni* to form biofilms [29, 30]. In this study, baicalin has a good anti-oxidative stress effect, so it is speculated that baicalin may participate in the formation of *A.lwoffii* biofilm by regulating *ahpC*. *mprA* (*mycobacterium* persistence regulator) is an important regulatory factor in two-component systems (TCSs), recognizing and responding to various environmental stimuli, including environmental stimuli that induce biofilm stress, and regulating the expression of growth factors under various conditions, among which it directly regulates two key stress response factors *sigB* (sigma B, a transcriptional regulator) and *sigE* [31, 32]. Therefore, strengthening the continuous research on TCSs is crucial for understanding the interrelated regulatory network of *A.lwoffii* surviving under environmental pressure and host infection. YidC protein is a membrane-located chaperone insertase that is universally conserved in all bacteria and traditionally studied in the context of membrane protein insertion and assembly [33]; Research has found that any deficiency of *yidC* will lead to changes in the structure and physical properties of EPS matrix produced by the deformed bacteria, ultimately damaging biofilm formation, reducing its mechanical stability and promoting its eradication [34, 35]. At the same time, combined with differential gene expression analysis, it was found that Rcs phosphorylation pathway participates in the regulation of many cell surface structures in intestinal bacteria. The sensor histidine kinase RcsC autophosphorylates and transfers phosphate to the response regulator RcsB through intermediate steps. Once RcsB is phosphorylated, it regulates gene expression, thereby inhibiting the expression of biofilm-forming gene *csgD* and leading to biofilm inhibition [36, 37]. Combined with this work, it is speculated that the drug target of baicalin may be related to the above genes, providing a theoretical basis for possible drug targets for biofilm associated infections (BAI) prevention and treatment.

Molecular docking is one of most commonly used techniques in the structure-based drug design approach. This technique is generally used to understand the interaction of ligand molecules binds to the specific active site of proteins and docking score of the ligand-receptor complex was analyzed by reference ligand score. M.Abinaya et al. found from the molecular docking analysis that the 3, 5, 7-Trihydroxyflavone can act as an anti-quorum sensing agent against *P.aeruginosa* and control the biofilm formation [38]. Vijayakumar et al. verified by Molecular docking analysis predicted the ability of hesperidin to interact with SarA and CrtM proteins involved in biofilm formation and staphyloxanthin production in MRSA [39]. To study the effect of the interactions between the 26 target proteins, which is related to the biofilm formation in *A.lwoffii*, with the baicalin was carried out the analysis of molecular docking model. In molecular docking, the lower the value of the score function, the better the affinity between the linker and the receptor [40]. As can be seen in Table 2 active ingredient showed negative binding free energy results at the active site of the protein, showing that baicalin has high affinity with the proteins. Therefore, this effectively support the baicalin can eradicate the preformed biofilm of *A.lwoffii.*

Trehalose is present in a large number of organisms and is a typical stress metabolite. It can form a unique protective film under extremely harsh conditions (such as drying, dehydration, and high osmotic pressure), protecting the structure of biomolecules from being destroyed, thereby maintaining the life characteristics of the organism [41, 42]. The genome of *Pseudomonas baumannii* encodes OtsB and OtsA. OtsA plays a key role in stress resistance and glucose metabolism, while the absence of *otsB* can affect the formation of trehalose. The genes *otsA* and *otsB* constitute an operon, and their expression is closely related to osmotic stress and stationary phase induction [43, 44]. This work found that the transcription levels of the *otsA* and *otsB* genes were upregulated by about 2 times and 4 times, respectively. It is speculated that this may be a self-protection mechanism caused by the bacterial stress response due to the intervention of baicalin in the biofilm bacteria of *A.lwoffii*. Moreover, through UV spectrophotometry, it was found that the amount of trehalose significantly decreased after the intervention of baicalin in the biofilm of *A.lwoffii*. This further indicates that trehalose may be involved in the formation of biofilms in *A.lwoffii*, and the specific mechanism needs to be further studied.

## Conclusion

This study evaluated the antibiofilm potential of baicalin against *A.lwoffii*. Baicalin revealed strong antibiofilm potential against *A.lwoffii*. RT-qPCR analysis showed that the down-regulation of genes (*yidC*,*rpiJ*, *liuE*) involved in biofilm formation, the up-regulated genes (*otsA*, *betB*, *mprA*, *rcsC-3*, *otsB*, *ahpC*) involved in oxidative phosphorylation and TCSs in *A.lwoffii* upon baicalin treatment. Molecular docking analysis predicted the ability of baicalin to bind the active sites of target proteins in *A.lwoffii*. Moreover, the content of trehalose decreased may be related to biofilm eradication. This study suggests that baicalin may be a potential antibiofilm agent, and the mechanism of the biofilm eradication of *A.lwoffii* needs to be further studied.

## Methods

### Bacterial strains,culture conditions

*A.lwoffii* strain was resuscitated, centrifuged at 400 × g for 10 min, the culture was expanded, then the supernatant was discarded and 8 mL sterile LB broth was added to resuspension. The optical density (OD) of 600 nm was adjusted to 0.1, and then diluted 100 times as the test bacterial solution.

### Biomass and biofilm bacterial assays

The biomass and viable bacteria of biofilms were assayed, as described previously with minor modifications [45]. The results demonstrated that the MIC value of *A.lwoffii* to baicalin was 2048 μg/mL. The final concentrations of baicalin were 1/2 MIC, MIC, 2 MIC, 4 MIC and 8 MIC μg/mL. Strains were cultured overnight at 37 °C in Luria Bertani (LB) broth, centrifuged, diluted to 0.1 optical density at 600 nm (*OD600*) and then diluted 100 times as the test bacterial solution. The biofilms were preformed by adding the test bacterial solution into 96-well plates and incubating at 37 °C for 24 h. Afterwards, the plates were washed three times with phosphate-buffered saline (PBS). The double dilutions ranging from 8 MIC to 1/2 MIC for baicalin was prepared with LB broth. Next, 100 μL of each concentration was added to the corresponding plate and incubated at 37 °C for 3 h. Briefly, the supernatant was discarded, and the cells were washed twice with sterile PBS. Fixing with 99% methanol for 10 min, followed by air drying, staining with 0.04% crystal violet solution for 20 min, and washing with sterile PBS. Then, 33% acetic acid was used to dissolve the bound crystal violet, and absorbance was measured at *OD*_*600*_ nm. To count the number of bacteria in the biofilm, 100 μL Triton X-100 was added to each well to disrupt the biofilm, followed by tenfold dilution and spreading on TSA plates, and the colonies were counted after 12 h at 37 °C.

### Confocal laser scanning microscopy (CLSM)

The morphological features of biofilms were observed by CLSM as described previously with some modifications [46]. In this experiment, 100 μL of the undiluted test bacterial solution (*OD600* = 0.1) was added to an 8-well chambered cover glass (1.5 Borosilicate glass, Lab-Tek II chambered coverglass). After incubation for 24 h at 37 °C, the biofilm was treated with baicalin at 37 °C for 3 h. Then, the biofilm was washed with 0.9% (wt/vol) NaCl and stained for 20 min in the dark at room temperature using a FilmtracerTM LIVE/DEADTM Biofilm Viability kit. After being rinsed with sterile water, the biofilm samples were imaged with a point-scanning confocal microscope, which was equipped with a Plan-Apochromat 63 × /1.40 oil objective lens. Signals were recorded using the green (SYTO9, excitation wavelength of 488 nm) and red (PI, excitation wavelength of 561 nm) channels. The three-dimensional (3D) image was constructed by superimposing multiple images with different Z values (z-stack). Images were acquired using ZEN (black edition) software. Four representative images were selected from each biofilm, and each experiment was repeated at least three times. The biofilm volume, area and fluorescence intensity were analyzed by BiofilmQ software [47].

### RNA-seq analysis

*A.lwoffii* biofilms were cultured in 6-well cell culture plates for 24 h. The experimental group was treated with 4 MIC of baicalin for 3 h, simultaneously set up a broth control group. Three independent repeat samples were collected from each experimental group and sent to the company for RNA-seq sequencing. The expression levels of genes were analyzed using DESeq software. The threshold of gene expression was represented by FPKM values (0.1 or 1), and this work only analyzed genes with FPKM > 1. Differential genes were subjected to KEGG pathway and GO analysis.

### Quantitative real-time PCR (RT-qPCR)

The RNA extraction and cDNA synthesis were performed using the method of Roudashti and Zhou et al. with some modifications [48, 49]. RNA was extracted from biofilms of drug groups constructed on 6-well plates. Briefly, biofilms were washed three times with pre-chilled PBS solution, and the wells were gently and thoroughly scraped, then collected in 1.5 mL centrifuge tubes. RNA extraction was performed using the Spin Column Bacteria Total RNA Purification Kit according to the manufacturer’s manual. The concentration and purity of the RNA samples of per group were detected by using a Spectrophotometer (μLite, BioDrop) at 260/230 nm and 260/280 nm, respectively. Reverse transcription was performed to obtain cDNA on the basis of the manufacturer’s manual at 37 °C for 15 min, 85 °C for 5 s. The cDNA samples were used for RT-qPCR detection.

RT-qPCR was used to measure the effect of 4 MIC baicalin on the expression of 8 differential genes most related to the biofilm of *A.lwoffii* using the primers listed in Table 3 (the 16 SrRNA, as the reference gene). The operationof RT-qPCR was carried out according to the instructions of the commercial kit (Takara, PrimeScriptTM RT reagent Kit (Perfect Real Time) in a 20 μL total reaction volume. The reaction steps proceeded as follows: 40 cycles of PCR were carried out with denaturation at 95 °C for 30 s, annealing at 95 °C for 5 s and extension at 60 °C for 30 s. Control and experimental groups were repeated three times. The relative expression levels of each target gene were detected by 2 ^−ΔΔCt^ method.

**Table 3:** Gene primer sequences

Gene	Forward primer(5’ → 3’)	Reverse primer(5’ → 3’)
*rcsC*_3	GCGCTATTTTCAGGAACAGACC	TCTGATTGGAACTCAGCGGAAA
*otsA*	TTTGCCAAGCATCTGAAACAGG	GGTTACGCATACCCAGTTTTCG
*otsB*	GGATTGTCTGACCCACTTTCAAG	AATGAGCTTGCTGGTGAATGTG
*yidC*	GTTGACTCAGGGTAATGGTGAAG	CAGTACCTGCAGGCACATTAAAG
*betB*	ATAAATACCTGGGGCGAATCTCC	GGTCTGGGTATATTGCTGCAAAG
*liuE*	GGTCTGGGTATATTGCTGCAAAG	GCCCATATTCGAGAGCAGGTAA
*ahpC_*	GTCCAACTGAATTAGGCGATCTTG	GAGGTATCGTGCCAAGCTTTATG
*rpiJ*	TTCCCGTGGCGTAACTGTAG	CAGCTTCTTTTGCATCGCGT
*mprA*	CGGGTTATGAACTGTGTGAATGG	GCCTTTGAGTTTGTCATCCAGAG
16 *SrRNA*	CAGCMGCCGCGGTAATWC	CCGTCAATTCMTTTRAGTTT

### Molecular docking

The amino acid sequences of proteins related to the biofilm of *A.lwoffii* were obtained from the protein database in NCBI (https://www.ncbi.nlm.nih.gov/). The homology model was generated and assessed using the Swiss model (https://swissmodel.expasy.org). The crystal structure of proteins was obtained from the protein database in PDB (https://www.rcsb.org/). Molecular docking was performed with the proteins as the receptor and the active ingredient as the ligand. AutoDockTools 1.5.7 software was applied to process proteins. PyMOL (https://www.pymol.org/) is an open source molecular modeling visualization software, which can analyze the binding situation of receptor proteins and ligands in molecular docking, and it can mark hydrogen bonds.The top 6 binding force compounds were imported into PyMOL for further visualization.

### Assays of trehalose content

Sodium alginate standard product was diluted with ddH_2_O water at a ratio, with concentrations of 0.1, 0.05, 0.025, 0.0125, 0.00625, 0.003125 mg/mL respectively. A volume of 0.25 mL standard solution and 1 mL working solution were added to an EP tube and boiled at 95 °C for 10 min. When the solution was cooled to room temperature, added the solution to the colorimetric dish and measured at *OD*_*620*_nm. Calculate the concentration of ΔA standard (ΔA standard = A standard—A blank), with the concentration of the standard tube (self-matching concentration, mg·mL-1) as the horizontal coordinate (X-axis), and the absorbance A standard (calculated ΔA standard) as the vertical coordinate (Y-axis), draw the standard curve and perform linear regression to analyze its results. Assays of trehalose content in *A.lwoffii* biofilm by baicalin. Cultivate *A.lwoffii* biofilm for 24 h, use 4 MIC baicalin for 3 h, and set a control group, Triton lyses biofilm, centrifuge to discard supernatant; add appropriate amount of extraction solution according to the instructions, ultrasonic crushing (ultrasonic for 3 s, interval for 10 s, repeat for 30 times, 200 W), stand for 1 h, centrifuge at 8000 g for 15 min, take supernatant to EP tube, boil at 95 °C for 10 min, cool to room temperature, take solution to colorimetric dish, measure *OD*_*620*_nm. Using the formula obtained from the drawn standard curve to calculate X value can measure the trehalose content of each group.

### Statistical analysis

All experiments were performed in at least three independent assays. The experimental data was processed using GraphPad Prism 8.0 and Microsoft Excel software, and analyzed using Student’s t-test and one-way analysis of variance. Significant differences are indicated as *(*P* < 0.05), **(*P* < 0.01), ***(*P* < 0.001), and ****(*P* < 0.0001).

### Supplementary Information

**Supplementary Material 1.**

## Data Availability

Data is provided within the manuscript or supplementary information files

## Abbreviations

EPS: Extracellular polymeric substances
TCM: Traditional chinese medicine
TCSs: Two-component systems
MIC: Minimum inhibitory concentration
CLSM: Confocal laser scanning microscopy
PI: Propidium iodide
FPKM: Fragments per kilobase million
KEGG: Kyoto encyclopedia of genes and genomes
GO: Gene ontology
qRT-PCR: Quantitative real-time PCR
TCRS: Two-component regulatory systems
BAI: Biofilm associated infections
LB: Luria bertani
PBS: Phosphate-buffered saline
